# The association between beverage consumption pattern and dental problems in Iranian adolescents: a cross sectional study

**DOI:** 10.1186/s12903-020-01065-y

**Published:** 2020-03-17

**Authors:** Naimeh Hasheminejad, Tayebeh Malek Mohammadi, Mohammad Reza Mahmoodi, Moein Barkam, Arash Shahravan

**Affiliations:** 1 Oral and Dental Disease Research Center and Kerman Social Determinants on Oral Health Research Center, Kerman University of Medical Sciences, Kerman, Iran; 2 Social Determinants of Health Research Center, Institute for Futures Studies in Health, Department of Dental Public Health, Kerman University of Medical Sciences, Kerman, Iran; 3 Physiology Research Center, Institute of Basic and Clinical Physiology & Department of Nutrition, Faculty of Health, Kerman University of Medical Sciences, Haft Bagh-E-Alavi Highway, Kerman, 7635111167 Iran; 4 Endodontology Research Center, Kerman University of Medical Sciences, Kerman, Iran

**Keywords:** Adolescents, Beverage, Dental caries, Tooth erosion

## Abstract

**Background:**

With regard to the increasing consumption rates of unhealthy beverages among adolescents, the main purpose of the present study was to determine the association between beverage intake pattern and dental caries and tooth erosion in this age group.

**Methods:**

A total sample of 600 adolescents was recruited in this study using a multistage cluster random sampling method in the city of Kerman, in the southeast of Iran, in 2017. Then, the Decayed, Missed and Filled Teeth (DMFT) index and the Tooth Wear Index (TWI) were registered for each participant. A Beverage Frequency Questionnaire was also employed to estimate typical beverage intake frequency. Correspondingly, negative binominal regression and logistic regression were performed to determine the independent variables associated with the DMFT index and the TWI.

**Results:**

The findings revealed that the highest consumed beverage in daily living was tea in both genders, followed by sweetened soft beverages, as well as milk and kefir/yogurt drink. The results of the DMFT index were also significantly different in participants that had never consumed milk compared with those who had used milk on a daily basis. Moreover, the DMFT index in participants who had never consumed sweetened soft beverages was 39%, less than those who had had a daily intake of such beverages. Also, the chance of tooth erosion for participants who had never used sweetened soft beverages was 94%, lower than that in daily consumers.

**Conclusions:**

The results of this study revealed that adolescents had an unhealthy beverage intake pattern. Furthermore, milk consumption was beneficial to dental caries, whereas use of soft drinks associated with more dental caries and tooth erosion.

## Introduction

Adolescence has been recognized as an important period of life in terms of food-related behaviors. During this period, adolescents spend more time with their friends and also make many food choices independently [1]. Avoiding healthy meals and consuming unfavorable sugar-containing beverages are frequent behaviors that impair health during this period [2]. Significant increase in soft drink consumption has been thus reported especially among adolescents [3, 4]. In this respect, a study demonstrated that almost half of calories from added sugars was related to beverages [5]. According to the related literature, higher consumption of sugar-containing beverages had resulted in obesity and lower use of nutritious food especially milk [4, 6].

Recently, concerns have been raised regarding dental caries and tooth erosion as other consequences of using high sugar-containing beverages [6, 7]. Basically, consumption of foods with high sugar content in liquid form increases incidence of dental caries [8].

On the other hand, there are beverages known for their protective characteristics against dental caries. For example, the buffering capacity of milk inhibits the process of dental caries [9]. Also, studies have reported that different components of green tea could be effective in reducing caries lesions by inhibiting biological activities of streptococci [10–12].

In this line, Gueilnckx et al. [6] had found that people of different countries could vary greatly in terms of consuming different types of drinks. Temperature and humidity could also affect type of beverage preferences in a region. Also, certain types of beverages could be consumed more in a country out of traditio. Likewise, different foods consumed in a country could influence beverage preferences of that region. However, very few researchers had assessed drinking pattern of adolescents in Iran and most studies had merely focused on sugar-containing drinks [3, 8, 13]. As many lifestyle behaviors are established during this period [14], it is of utmost importance to identify beverage intake pattern among adolescents in Iran. Also, if beneficial and harmful effects of different beverages are identified, it would assist future oral health programs to substitute harmful beverages with more beneficial ones.

Accordingly, the main purpose of this study was to find whether there were significant differences between consumption of different types of beverages and dental caries and tooth erosion or not. Furthermore, this study sought to estimate and compare frequency of all types of accessible beverages among adolescents of both genders in the city of Kerman, in the southeast of Iran.

## Methods

This study was an analytical cross-sectional research conducted in the city of Kerman in the southeast of Iran. Based on the mean DMFT index and standard deviation (SD) related to adolescents aged 12 years old in the city of Kerman, published by Iranian National Health Survey [15], considering α = 0.05, d = 0.02, and SD = 0.24, a sample size of 600 adolescents (300 females and 300 males) were randomly selected to participate in the study. A multistage cluster random sampling method was also used to select these individuals from eight schools (4 schools for girls and 4 schools for boys). This sampling method was composed of two methods, cluster random sampling and systematic random sampling.

At first, in order to select the clusters in each of the four municipality zones of the city of Kerman, two schools were randomly selected (one school for girls and one school for boys) by a statistical consultant using the mapping guideline of the schools in the Department of Education. In each school, a total of 75 adolescents aged 12–15 years old were selected using systematic random sampling method through the booklet for pupil identification by ascending order of grading. After selecting the adolescents, they were invited to participate in the study. A total of 600 written informed consents were also obtained from parents of children, for their participation in the study. Overall, 20 participants were excluded due to having orthodontic devices and systemic diseases, who were then replaced by other adolescents using the sampling strategy explained earlier.

The study proposal was approved by the Ethics Committees (KMU-0138) of the Vice-Chancellor’s Office for Research at Kerman University of Medical Sciences and the data collection was carried out from December 2017 until April 2018.

The objectives and the course of the study were explained and written informed consent was also obtained from children’s parents before data collection. Adolescents with systemic diseases such as bleeding, epilepsy, and eating disorders; those undergoing orthodontic treatments (wearing braces), and adolescents with a history of radiotherapy or chemotherapy were additionally excluded from the study. In case of any exclusion, substitution was done through the sampling method. All the participants were interviewed one-by-one in a separate room. A questionnaire comprised of several sections including information regarding socioeconomic status, demographic characteristics, and tooth brushing status was also completed for each participant.

The participants were asked about frequency of using different types of beverages over the past 3 months as they recalled. A researcher-designed beverage frequency questionnaire (validated but not published) containing a complete list of available beverages in Iran, was then completed for each participant. This questionnaire was designed according to the modified National Health and Nutrition Examination Survey (NHANES) Food Frequency Questionnaire (FFQ) [16] using a scale of once in 3 months or less, once a month or less, 2–3 times a month, 1–2 times a week, 3–4 times a week, 5–6 times a week, once a day, 2–3 times a day, 4–5 times a day, and 6 times a day or more. The validity and the reliability of the questionnaire was also confirmed by a group of five nutrition experts using the Delphi method (Cronbach’s alpha = 0.72).

In order to ensure participants’ responsiveness, a researcher-designed booklet containing pictures of beverages was shown to the participants and the questionnaire was completed simultaneously. Also, pictures of common cups and glasses with defined volumes were shown to each adolescent with the purpose of calculating frequency according to standard volumes.

Dental examination was carried out by an examiner trained and calibrated by the World Health Organization (WHO) Oral Health Surveys Basic Methods [17] and other dental indices (weighted Cohen’s kappa = 80%). All the participants were examined on a chair in a comfortable position with a head light, a plane mirror, and the Community Periodontal Index (CPI) probe, used for checking dental status. First, the Plaque Index (PLI) developed by Silness and Loe was utilized to record the plaque [18]. Then, the teeth were cleaned using a sterilized gauze and cotton to determine the DMFT index according to the procedures recommended by the WHO [17]. Finally, the tooth wear index (TWI) designed by Smith and Knight [19] was employed to determine tooth erosion.

### Statistical analysis

In order to simplify the expression and the interpretation of beverage consumption frequencies, they were merged and classified into four groups: (a) “never”, (b) integration of once in 3 months or less, once a month or less, and 2–3 times a month under “occasionally”, (c) integration of 1–2 times a week, 3–4 times a week, and 5–6 times a week under “weekly”, and (d) integration of once a day, 2–3 times a day, 4–5 times a day, and 6 times a day or more under “daily”. Also, the number of beverage groups was high in proportion to the sample size. Therefore, similar beverages were merged and classified into seven groups as follows; Group (1): milk beverages, Group (2): milk products such as soy milk, chocolate milk, and other flavored milk, Group (3): kefir/yogurt drink, Group (4): tea beverages such as black tea, green tea, and herbal tea, Group (5): sweetened soft beverages e.g. non-alcoholic beer, carbonated drinks, diet drinks, energy drinks, artificial fruit juice, lemonades, and distilled essential oil syrups, Group (6): natural fruit juice and fruit nectar, and Group (7): other hot beverages such as coffee, cappuccino, Nescafe, and hot chocolate.

In addition, Stata (version 11) (Stata Corp, 2009, Stata Statistical Software: Release 11. College Station, TX: Stata Corp LP) was used as the statistical software to analyze the data. Also, Chi-square tests were utilized to find the relationship between beverage intake and demographic variables.

Negative binominal regression was further performed to determine the independent variables associated with the DMFT index (count variable) using a backward modeling strategy. Also, the TWI was considered as a binary variable (those with/without tooth wear). Using a backward modeling strategy, logistic regression models were similarly employed to determine the variables associated with the TWI. For both modeling strategies (i.e. negative binominal and logistic regression), all the variables were initially entered into the model one-by-one, then, the variable with the least *p*-value was put into the model and this process continued into four models until the complete model was created. Model 1 was adjusted for PLI; Model 2 was adjusted for PLI and seven beverage consumption groups categorized into four classes of consumption frequency (never, occasionally, weekly, and daily); Model 3 was modified for PLI, tooth brushing status, and gender; and Model 4 was set for all variables. Daily consumption frequency was also used as the reference variable. Moreover, incidence risk ratio (IRR) and odds ratio (OR) were reported for other three consumption frequencies.

The significance level was considered by *p* < 0.05. Estimates were further reported at 95% confidence interval (CI).

## Results

The results of this study revealed that 50% (*n* = 300) of the adolescents were male. The mean age of these individuals was also 13.2 ± 0.7 years and the mean PLI was 1.4 ± 0.7. Socioeconomic status and demographic characteristics of the participants are shown in Table 1.

**Table 1:** Baseline socio-demographic characteristics of the participants

	Boy (*N* = 300)	Girl (N = 300)	*P*-value
Age, years; mean ± SD	13.41 ± 0.76	13.15 ± 0.75	< 0.0001
Father Educational level; N (%)			0.001^a^
Illiterate	25 (8.3)	24 (8.0)	
Middle school	118 (39.3)	87 (29.0)	
Diploma	91 (30.3)	96 (32.0)	
Upper Diploma	16 (5.3)	23 (7.7)	
Bachelor’s degree	11 (3.7)	36 (12.0)	
Post graduate	15 (5.0)	23 (7.7)	
Missing	24 (8.1)	11 (3.7)	
Total	300 (100)	300 (100)	
Mother Educational level; N (%)			0.019^a^
Illiterate	17 (5.7)	17 (5.7)	
Middle school	87 (29.0)	79 (26.3)	
Diploma	126 (42.0)	113 (37.7)	
Upper Diploma	27 (9.0)	24 (8.0)	
Bachelor’s degree	14 (4.7)	37 (12.3)	
Post graduate	12 (4.0)	20 (6.7)	
Missing	17 (5.6)	10 (3.3)	
Total	300 (100)	300 (100)	
Tooth-brush; N (%)			< 0.0001^a^
Less than once a day	205 (68.3)	84 (28.1)	
At least once a day	95 (31.7)	215 (71.9)	

^a^Statistically significant

Generally, 42% of the participants had consumed tea beverages in daily living but 18% of them had not had it over the past 3 months. Sweetened soft beverages had been also consumed by 25.6% of the participants; however, 1.8% of these individuals had not used them during the past 3 months. Furthermore, 23.3% of the participants had consumed milk at least once a day, but 9.2% of them had not used this beverage at all in the past 3 months.

Table 2 shows consumption frequency for each beverage group after integrating and classifying 19 beverage groups. It was observed that male and female participants were statistically different only for the consumption of kefir/yogurt drink and other hot beverages.

**Table 2:** The frequency (percentage) of participants according to categories of beverage consumption and gender

Group	Gender	Never	Occasionally	Weekly	Daily	*P*-value
Milk beverage	male	27 (49.1)	27 (38.0)	176 (52.7)	70 (50.0)	0.168
	female	28 (50.9)	44 (62.0)	158 (47.3)	70 (50.0)	
Milk derivatives	male	61 (56.5)	50 (40.0)	164 (51.7)	25 (50.0)	0.066
	female	47 (43.5)	75 (60.0)	153 (48.3)	25 (50.0)	
Yogurt drinks	male	50 (62.5)	40 (39.2)	169 (53.1)	41 (41.0)	0.003^a^
	female	30 (37.5)	62 (60.8)	149 (46.9)	59 (59.0)	
Tea beverages	male	61 (55.5)	11 (31.4)	102 (50.2)	126 (50.0)	0.105
	female	49 (44.5)	24 (68.6)	101 (49.8)	126 (50.0)	
Sweetened soft beverages	male	8 (72.7)	26 (40.6)	195 (52.6)	71 (46.1)	0.092
	female	3 (27.3)	38 (59.4)	176 (47.4)	83 (53.9)	
Natural fruit juice	male	65 (60.7)	51 (46.8)	160 (48.5)	24 (44.4)	0.095
	female	42 (39.3)	58 (53.2)	170 (51.5)	30 (55.6)	
Other hot beverages	male	141 (54.0)	53 (43.4)	91 (53.5)	15 (31.9)	0.013^a^
	female	120 (46.0)	69 (56.6)	79 (46.5)	32 (68.1)	

^a^ statistically significant

The daily consumption of tea, milk, and milk products was similar between males and females. However, the daily consumption of kefir/yogurt drink, sweetened soft beverages, natural fruit juice, and other hot beverages was higher among female participants. Overall, the highest consumed beverage in daily living was tea, followed by sweetened soft beverages, milk, and kefir/yogurt drink.

Sweetened soft beverages were also the most consumed beverage group on a weekly basis. Also, the number of males who had used milk, milk products, kefir/yogurt drink, sweetened soft beverages, and other hot beverages on a weekly basis was more than females. The number of male and female participants who had used tea beverages weekly was equal. Meanwhile, the number of females who had consumed natural fruit juice on a weekly basis was more than males.

The mean DMFT index of the participants was 4.0 ± 3.0. Among the adolescents, 84.5% had at least one decayed tooth and only 15.5% of them were caries-free. Also, 8% of them had at least one missed tooth and 17.5% of these individuals had at least one filled tooth. The mean decayed, missed, and filled teeth were 3.6, 1.1 and 0.3; respectively.

The results of negative binominal regression and backward modeling of the DMFT index (count variable) based on the independent variables of PLI, consumption frequency of seven beverage groups, gender (male/female), and tooth brushing status (less than once a day and at least once a day) are presented in Table 3.

**Table 3:** Modeling DMFT based on Negative Binomial regression model^b^

Parameter and confounders	Model 1^c^ IRR (95% CI)	Model 2^d^ IRR (95% CI)	Model 3^e^ IRR (95% CI)	Model 4^f^ IRR (95% CI)	*P*-value
Plaque index	1.23 (1.13, 1.34)^a^	1.23 (1.13, 1.34)^a^	1.27 (1.15, 1.39)^a^	1.25 (1.14, 1.37)^a^	< 0.001
Milk beverages					0.006
Occasionally		1.10 (0.94, 1.30)		1.11 (0.95, 1.31)	
Weekly		1.38 (1.09, 1.74)^a^		1.38 (1.09, 1.74)^a^	
Never		1.31 (1.03, 1.67)^a^		1.34 (1.05, 1.71)^a^	
Milk derivatives					0.760
Occasionally		0.93 (0.74, 1.18)		0.93 (0.73, 1.17)	
Weekly		0.92 (0.70, 1.20)		0.92 (0.70, 1.20)	
Never		0.94 (0.71, 1.23)		0.94 (0.72, 1.24)	
Yogurt drinks					0.121
Occasionally		1.08 (0.90, 1.30)		1.08 (0.90, 1.30)	
Weekly		1.14 (0.91, 1.42)		1.13 (0.91, 1.41)	
Never		1.15 (0.91, 1.45)		1.15 (0.91, 1.45)	
Tea beverages					0.759
Occasionally		1.04 (0.90, 1.20)		1.03 (0.90, 1.19)	
Weekly		1.14 (0.87, 1.50)		1.12 (0.85, 1.48)	
Never		0.96 (0.80, 1.14)		0.97 (0.81, 1.16)	
Sweetened soft beverages					0.127
Occasionally		0.85 (0.73, 0.99)^a^		0. 85 (0.74, 1.0)	
Weekly		0.90 (0.71,1.15)		0.91 (0.71, 1.15)	
Never		0.61 (0.37,0.99)^a^		0.64 (0.39, 1.06)	
Natural fruit juice					0.505
Occasionally		1.21 (0.96, 1.53)		1.21 (0.96, 1.53)	
Weekly		1.27 (0.97, 1.67)		1.27 (0.97, 1.66)	
Never		1.01 (0.77, 1.31)		1.00 (0.76, 1.31)	
Other hot beverages					0.882
Occasionally		1.03 (0.80, 1.33)		1.02 (0.79, 1.32)	
Weekly		0.95 (0.72, 1.25)		0.95 (0.72, 1.25)	
Never		1.04 (0.81, 1.33)		1.03 (0.80, 1.32)	
Gender			0.99 (0.86, 1.14)	0.96 (0.83, 1.10)	0.727
Tooth brush			1.11 (0.96, 1.28)	1.10 (0.95, 1.28)	0.094

^a^Statistically significant

^b^Daily consumption was used as reference variable

^c^Model 1 was adjusted for plaque index

^d^Model 2 was adjusted for plaque index and 7 groups of beverage

^e^Model 3 was adjusted for plaque index, tooth brushing status and gender

^f^Model 4 was adjusted for all variables

The results of the DMFT index were significantly different in the participants that had never consumed milk compared with those who had used milk on a daily basis (*p* = 0.006). Although the results of the DMFT index were significantly different in the participants that had never consumed or had occasionally used sweetened soft beverages compared with its daily consumption in Model 2, the association was not significant in the final model (*p* = 0.127). According to Model 2, the results of the DMFT index of the participants who had never consumed sweetened soft beverages was 39%, less than those who had consumed them daily. Also, the participants who had consumed sweetened soft beverages occasionally had obtained 15%, lower DMFT index score compared with daily consumers. Model 3 was adjusted for PLI, tooth brushing status, and gender. The PLI was also the only significant variable in this model.

Finally, all the variables were adjusted in Model 4 and the PLI, weekly milk consumption, and never milk consumption were considered as the significant variables. Tooth brushing status and gender also made a slight change to the significance of the model. It seemed that Model 2 was the right one to determine the variables associated with the DMFT index. Overall PLI and milk consumption frequency were significantly associated with the DMFT index. Table 4 shows logistic regression modeling for the TWI.

**Table 4:** Modeling Tooth Wear Index based on logistic regression model^b^

Parameter and confounders	Model 1^c^ IRR (95% CI)	Model 2^d^ IRR (95% CI)	Model 3^e^ IRR (95% CI)	Model 4^f^ IRR (95% CI)	*P*-value
Plaque index	1.20 (0.80, 1.80)	1.13 (0.72, 1.77)	1.15 (0.74, 1.81)	1.06 (0.65, 1.73)	0.810
Milk beverages					0.707
Occasionally		0.86 (0.37, 2.02)		0.86 (0.36, 2.04)	
Weekly		1.59 (0.52, 4.87)		1.66 (0.54, 5.12)	
Never		0.50 (0.12, 2.06)		0.47 (0.11, 2.01)	
Milk derivatives					0.302
Occasionally		0.86 (0.28, 2.63)		0.88 (0.28, 2.71)	
Weekly		1.01 (0.28, 2.63)		1.03 (0.28, 2.71)	
never		1..43 (0.38, 5.37)		1.49 (0.39, 5.65)	
Yogurt drinks					0.428
Occasionally		0.94 (0.36, 2.46)		0.93 (0.35, 2.45)	
Weekly		1.76 (0.22, 2.57)		0.76 (0.22, 2.59)	
never		1.61 (0.55, 4.67)		1.58 (0.53, 4.72)	
Tea beverages					0.147
Occasionally		1.21 (0.54, 2.70)		1.22 (0.54, 2.75)	
Weekly		2.43 (0.67, 8.21)		2.46 (0.69, 8.75)	
never		1.91 (0.80, 4.57)		1.80 (0.74, 4.40)	
Sweetened soft beverages					< 0.0001
Occasionally		0.27 (0.13, 0.55)^a^		0.27 (0.13, 0.55)^a^	
Weekly		0.06 (0.01, 0.51)		0.06 (0.01, 0.50)^a^	
never		Empty		Empty	
Natural fruit juice					0.73
Occasionally		1.75 (0.46, 6.67)		1.77 (0.47, 6.90)	
Weekly		2.99 (0.69, 12.98)		3.02 (0.69, 13.22)	
never		3.58 (0.86, 14.97)		3.58 (0.85, 15.16)	
Other hot beverages					0.730
Occasionally		1.29 (0.37, 4.49)		1.26 (0.36, 4.43)	
Weekly		1.17 (0.30, 4.55)		1.15 (0.29,4.47)	
never		0.98 (0.29, 3.32)		0.96 (0.28,3.29)	
Gender			0.98 (0.49, 1.97)	0.97 (0.46, 2.06)	0.878
Tooth brush			0.87 (0.43, 1.75)	0.81 (0.37, 1.76)	0.806

^a^Statistically significant

^b^Daily consumption was used as reference variable

^c^Model 1 was adjusted for plaque index

^d^Model 2 was adjusted for plaque index and 7 groups of beverage

^e^Model 3 was adjusted for plaque index, tooth brushing status and gender

^f^Model 4 was adjusted for all variables

Sweetened soft beverages were significantly associated with the TWI in Models 2 and 4. For the participants who had occasionally consumed sweetened soft beverages, the chance of tooth wear was 73% (1–0.27 = 0.73) less than that in daily consumers (*P* < 0.001). Also, the chance of tooth wear for the participants who had never consumed sweetened soft beverages was 94% (1–0.06 = 0.94) less than daily consumers (*p* < 0.001). Tooth brushing status and gender had not correspondingly changed the significance of the model. Therefore, Model 2 seemed to be suitable. Finally, sweetened soft beverages were the only significant variable in this model.

## Discussion

This study was the first one in Iran, to the best of authors’ knowledge, using a complete assessment of beverage intake pattern to identify the association between dental caries and tooth erosion and beverage consumption among adolescents. The findings revealed that a number of commonly used beverages such as sweetened soft beverages and carbonated ones could be harmful to teeth. Conversely, milk was found to have protective effects on dental caries.

In order to limit any potential bias, a trained interviewer recorded beverage consumption frequency through a face-to-face interview, making sure that the beverage type and frequency had been recorded as precise as possible. Also, pictures of prevalent cups and glasses with specified volumes were presented, so that beverage consumption was standardized and comparable.

After controlling for the PLI, the findings suggested that the adolescents who had consumed sweetened soft beverages on a daily basis had a worse DMFT index score compared with those who had not used these beverages over the past 3 months. In this study, sweetened soft beverages consisted of carbonated beverages, artificial fruit juice, and non-alcoholic beer. The sugar and acid content of these beverages was probably the cause of dental caries and tooth erosion. According to Palacios et al. [20], sweetened beverages and fruit juices were the main sources of sugars in diets of children and adolescents leading to high pH drops of dental plaque, thus responsible for high prevalence of dental problems. Other studies had also highlighted caries proceeding effect of carbonated drinks which could decrease surface hardness of tooth enamel and make tooth more susceptible to caries [21, 22].

Similar to other studies, the only beverage group associated with tooth wear was sweetened soft beverages. Salas et al. [23] as well as Kannan et al. [24] had reported higher tooth wear among adolescents who had consumed high amounts of carbonated beverages. In a research by Pineda et al. [7], lower consumption of milk and milk products had been registered in addition to higher use of acidic beverages among adolescents who had tooth erosion [7]. Although consumption frequency of acidic beverages seemed to be an important etiology of tooth erosion, other factors such as swishing or holding the drink in mouth or brushing teeth immediately after the consumption of acidic beverages had been also mentioned as factors contributing to tooth erosion [21]. Therefore, future studies considering all these factors would help better understand the nature of tooth erosions related to consumption of acidic beverages.

The results of the DMFT index of the adolescents who had a daily consumption of milk was significantly lower than those who had not consumed milk over the past 3 months. Compared with sucrose, glucose, and fructose, milk sugar (lactose) causes a smaller plaque pH drop [20]. Therefore, most studies seemed to present milk as a beverage which was less cariogenic than other drinks (not an anti-cariogenic one) [20, 25]. Nevertheless, there are studies confirming that milk products could prevent caries to some extent, attributing it to different characteristics of milk such as its fat and protein content [23, 26, 27]. Regarding the effect of milk on dental decay, evidence supports the anti-cariogenic characteristics of milk, yet the design and procedure of studies vary greatly making it hard to come to a definite conclusion.

The findings of the present study showed that adolescents’ daily use of tea was higher than that of other beverages. In both genders, the daily consumption of sweetened soft beverages was more than milk. Only one in four adolescents had reported daily milk consumption and about one out of ten adolescents had not consumed milk during the past 3 months. One previous study in Iran had similarly revealed even lower percentage of adolescents who had consumed milk on a daily basis [28]. In the study in Sistan and Bluchestan province, Iran, nearly nine in ten adolescents had daily consumption of tea and three out of four had consumed sweetened soft beverages every day [28]. In the present study, less than half of the participants had used tea on a daily basis and various types of soft drink intake had been extremely lower compared with the findings in the study conducted in Sistan and Bluchestan province [28]. Apparent cultural differences in populations residing in diverse regions could be the reason for such a difference. Although information concerning the consumption frequency of different beverages is limited in Iran, all articles seem to focus on unhealthy drinking habits of adolescents.

The findings of this study revealed that approximately 12% of the adolescents had a daily consumption of carbonated beverages. Also, diet and energy drinks were not popular among the participants. Contrary to the results of the present study, nearly half of the American adolescents had daily intake of soft drinks and one out of five had daily consumption of energy drinks. Moreover, one in every five adolescents had diet soft drink consumption on a daily basis [29]. Although soft drinks are known to be extremely popular among adolescents from all over the world, it did not seem to be as popular as other countries in the present study. Lower socioeconomic status and lower soft drink availability at schools could be the main reason for such a discrepancy [30].

On the basis of the study findings, female participants had consumed sweetened soft beverages and natural fruit juice more than males. A number of studies had reported that males had frequently used unhealthy drinks such as carbonated beverages and sugar-containing ones [6, 30]. Whereas, another researcher had reported that males had higher consumption of all types of beverages even milk [29]. Due to controversies regarding gender differences in beverage consumption, it was not possible to compare the results.

In this region, the percentage of caries-free adolescents was extremely low (15.5%); therefore, factors associated with dental caries should be eliminated as much as possible. Sweetened soft beverages as an important factor leading to caries should be accordingly limited and milk consumption should be encouraged. Many studies had suggested that increased intake of healthier beverages especially milk could decrease the intake of sweetened soft beverages [31, 32]. The results of other studies had further reported that the availability of different snacks or beverages at home or schools could result in their higher intake [33–36]. Therefore, parents and school authorities need to be aware of oral health consequences related to sweetened soft beverage consumption and interventions towards lower availability of soft beverages at home or schools and recommending increased availability of milk. Likewise, public health efforts should prohibit excessive marketing of soft drinks and children’s access to such drinks should be limited. Finally, the findings of the present study emphasized the role of oral health on prevention of dental caries.

There were several limitations to the present study. As this study was a cross-sectional research, no causal relationship could be interpreted. Water consumption was not also assessed and there was lack of data regarding consumed sugar with tea, coffee, lemonade, and distilled essential oil syrup by adolescents. The other limitation was data collection time that was restricted to December 2017 until April 2018. Although the consumption of some drinks depends on the season of the year, use of beverages could have a long lasting effect on oral health. Therefore, this was not very noticeable because the beverage frequency questionnaire could predict beverage consumption for at least 1 year. Finally, a possible recall bias might have affected the results of the study. Based on the findings of this study, the use of tea as a daily beverage is popular in Iranian adolescents and future studies concentrating on the amount of sugar used with tea would be helpful. Also assessment of daily water consumption and association with intake of harmful beverages would also be beneficial in conducting population health programs.

## Conclusion

Iranian adolescents in the current study area had an unhealthy beverage consumption pattern and the findings revealed a possible positive effect of milk intake on dental caries; however, sweetened soft beverages were associated with a more severe experience of dental caries and tooth erosion. Therefore, these beverages should be reduced from children and adolescents’ daily diets as much as possible. Instead, adding milk to their diet might be more beneficial.

## Data Availability

The datasets used and/or analyzed during the current study are available from the corresponding author on reasonable request.

## Abbreviations

DMFT: Decayed, missed, filled teeth
TWI: Tooth wear index
KMU: Kerman Medical University
NHANES: National Health and Nutrition Examination Survey
CPI: Community periodontal index
PLI: Plaque index
